# Quality Improvement Project to Improve Communication with Patients in Relation to Section 136 (s136) Detention in St Mary’s Hospital A&E

**DOI:** 10.1192/j.eurpsy.2023.1026

**Published:** 2023-07-19

**Authors:** S. K. Ghaznavi, I. Ayeni, P. Kulkarni

**Affiliations:** 1 Liaison Psychiatry, Imperial College Healthcare NHS Trust; 2 Liaison Psychiatry, CNWL NHS Trust, London, United Kingdom

## Abstract

**Introduction:**

We have observed an increase in the number of patients detained under s136 of the Mental Health Act (MHA) within our emergency department. Patients are often transferred to the emergency department as an alternative place of safety. Many of these patients are provided very little information about the detention and the process of assessment. Best practice states that all patients should be provided written information. Currently, there is no such written information available with our emergency department or within the psychiatric liaison team in St Mary’s Hospital.

**Objectives:**

We aim to produce a user friendly information leaflet which will be distributed to all patients bought into the A&E department under s136. We aim to improve provision of written information from 0% to 50%.

**Methods:**

We aim to complete this project in three stages:

Stage 1: involvement of service users using the trust Expert by Experience Forum. We will co-produce a clear and user friendly leaflet detailing information pertaining to s136 and the process of assessment.

Stage 2: Once the leaflet is finalised, we will involve colleagues within the emergency department to ensure that there is an effective system which will ensure adequate distribution of the leaflet.

Stage 3: Once the leaflet is incorporated into our clinical practice, we will audit its distribution.

**Results:**

We will upload the finalised leaflet. We will also assess a random selection of notes from November 2022 to March 2023 to assess % of patients provided with written information.

**Conclusions:**

This project will result in co-production of an information leaflet for patients detained under s136 of the MHA. As such, we will aim to inform and improve the process for patients presenting to St Mary’s Hospital under the MHA.

**Disclosure of Interest:**

None Declared

